# Testing the Bidirectional Associations of Mobile Phone Addiction Behaviors With Mental Distress, Sleep Disturbances, and Sleep Patterns: A One-Year Prospective Study Among Chinese College Students

**DOI:** 10.3389/fpsyt.2020.00634

**Published:** 2020-07-17

**Authors:** Yinzhi Kang, Shuai Liu, Lulu Yang, Bixia Xu, Lianhong Lin, Likai Xie, Wanling Zhang, Jihui Zhang, Bin Zhang

**Affiliations:** ^1^ Department of Psychiatry, Nanfang Hospital, Southern Medical University, Guangzhou, China; ^2^ Guangdong-Hong Kong-Macao Greater Bay Area Center for Brian Science and Brain-Inspired Intelligence, Guangzhou, China; ^3^ Department of Internal Medicine, Zhaoqing Medical College, Zhaoqing, China; ^4^ Department of Psychiatry, Faculty of Medicine, Chinese University of Hong Kong, Hong Kong, Hong Kong

**Keywords:** Chinese college students, mobile phone addiction behaviors, mental distress, sleep disturbance, sleep patterns

## Abstract

**Background:**

Mobile phone addiction behaviors (MPAB) are extensively associated with several mental and sleep problems. Only a limited number of bidirectional longitudinal papers have focused on this field. This study aimed to examine the bidirectional associations of MPAB with mental distress, sleep disturbances, and sleep patterns.

**Methods:**

A total of 940 and 902 (response rate: 95.9%) students participated at baseline and one-year follow-up, respectively. Self-reported severity of mobile phone addiction was measured using Mobile Phone Involvement Questionnaire (MPIQ). Mental distress was evaluated by using Beck Depression Inventory (BDI) and Zung Self-Rating Anxiety Scale (SAS). Sleep disturbances were assessed by using Insomnia Severity Index (ISI), Pittsburgh Sleep Quality Index (PSQI), and Epworth Sleepiness Scale (ESS). Sleep patterns were evaluated by using reduced Morningness-Eveningness Questionnaire (rMEQ), weekday sleep duration, and weekend sleep duration.

**Results:**

Cross-lagged analyses revealed a higher total score of BDI, SAS, and ISI predicted a greater likelihood of subsequent MPAB, but not vice versa. We found the bidirectional longitudinal relationships between MPAB and the total score of PSQI and ESS. Besides, a higher score of MPIQ at baseline predicts a subsequent lower total score of rMEQ and shorter weekday sleep duration.

**Conclusions:**

The current study expands our understanding of causal relationships of MPAB with mental distress, sleep disturbances, and sleep patterns.

## Introduction 

Mobile phone has become an indispensable tool in people’s daily lives (1). However, uncontrolled or excessive use of mobile phones has arisen and is extensively associated with several physical and psychological disturbances (2). Mobile phone addiction (MPA) as a new concept aroused a wide range of interests (3–7).

MPA, a subset of technological addictions, which is defined as a behavioral addiction that involves human-machine interaction and is non-chemical (8). It was also named problematic mobile phone use, mobile phone dependence and smartphone addiction, but all highlight compulsive use, craving, tolerance, withdrawal, and functional impairment as key characteristics (8, 9). Further, MPA is considered as a generalized Internet addiction. In the 44th China Statistical Report on Internet Development, the percentage of Internet users using mobile phones reached 99.3% (10). Mobile phone has been irreplaceable devices for people with Internet addiction due to various use of Internet-based applications, such as mobile phone social media app, gambling, and gaming (11). In addition, some studies have found that adolescents with Internet addiction have high rates of MPA (12). Meanwhile, the portability and accessibility of mobile phones bring it with different characteristic, which can be distinguished from internet addiction (13).

MPA has shown that problematic mobile phone use has moderate and positive associations with depression severity and small to moderate associations with anxiety (4). Accredited theoretical frameworks for explaining the longitudinal relationship conceptualized mental distress driving MPA, rather than the other way around (4, 14). Especially, I-PACE model proposes that psychopathological features (e.g., depression, anxiety) and other variables (e.g., personality traits, dysfunctional personality traits) could be considered as factors representing predispositions of excessive internet use (such as MPA) (14). A mass of cross-sectional researches were conducted based on above theoretical framework. At present, only two studies have used prospective designs to explore the changes in MPA and the psychiatric symptoms over time, and one of them pointed out the reciprocal relationship between MPA and depressive symptom (15, 16). The exploration in the field still has limitations restricted by a relatively small sample size and a single aspect of mental distress concerned. More studies are needed to illuminate the cause and effect between them.

Furthermore, sleep disturbances are common problems in modern society. Most literature regarding mobile phone use and sleep problems focused on frequency, duration and specific time (e.g., bedtime, after light out) of use (17–21). It is reported that mobile device use was associated with at least one sleep outcome, such as insomnia, excessive daytime sleepiness, poor sleep quality, short sleep duration, and eveningness-chronotype. In addition, Liu (22) pointed out the vicious circle between excessive mobile phone use and sleep and/or mental health problems (22). However, only a limited number of literature have examined the potential effects between MPA severity and sleep problems, and all of them are cross-sectional studies, which are also confined to sample size or selection of statistical methods (7, 23–25).

In this study, we hypothesized that there are bidirectional relationships of MPAB with mental and sleep problems. Thus, the study among Chinese young adults was conducted and correlated variables were investigated in detail, such as mental distress, sleep disturbances, and sleep patterns. The aim was to further test the potential reciprocal associations of MPAB with them, respectively.

## Methods

### Participants

This is a one-year prospective study. We recruited 1050 freshmen aged 19 – 21 years from a medical college in Zhaoqing City, Guangdong, China in June of 2017 at baseline. The questionnaires were open-accessible for them through the internet platform of the college (https://www.welearning.net.cn/). The participants were requested to finish the task in their spare time. Nine hundred forty-four (89.5%) students had a valid response at baseline. At follow-up, 902 out of 940 (95.9%) responded with valid data in June of 2018. Informed consent has been obtained from every participant prior to two questionnaire surveys. This study has been approved by the institutional research ethics committee.

### Measures

#### Socio-Demographics and Living Habits

The variables examined in the socio-demographic section included gender and age of the participants, whether they are only child or not, their parents’ education level (bachelor degree or higher), whether their parents are migrant workers or not, and their family income (< 5,000 yuan/month or ≥ 5,000 yuan/month). The lifestyle factors included: Body-Mass Index (BMI, ≥25 kg/m2 or <;25 kg/m2), habitual napping, habitual snoring, boarding in school, daily duration of TV or internet (> 3 h/day or ≤ 3 h/day), tobacco smoking, alcohol drinking, chronic medical conditions, perceived study pressure (high or low), perceived interest in study (high or low).

#### Mobile Phone Involvement Questionnaire (MPIQ)

The questionnaire is a self-rating scale including 8 items on mobile phone involvement, based on Brown’s behavioral addiction components and then developed by Walsh et al. (26, 27). The questionnaire was designed to measure the qualitative descriptions of mobile phone addiction behaviors, such as withdrawal, cognitive and behavioral salience, euphoria, loss of control, relapse and reinstatement. Responses ranged from 1 (strongly disagree) to 7 (strongly agree). Items were summed up and the higher score is construed as the stronger dependency on mobile phone.

#### Beck Depression Inventory (BDI)

The inventory is comprised of 21 items that evaluated the severity of depression symptoms in the past week. Each item provides four points. The degree of depression is indicated by the total score, ranging from 0 to 63. Higher total scores indicate greater depression (28). BDI has been translated and well-validated in Chinese sample, the Cronbach’s alpha was 0.89 (29).

#### Zung Self-Rating Anxiety Scale (SAS)

The scale, originally developed in 1971, measures one’s experiencing anxiety symptoms in the past week (30). It consists of 20 self-rated questions, with each item rated on a 4-point scale (1 = never or a little of the time, 4 = most of the time). The total score was acquired by multiplying the raw score by 1.25. The validity of Chinese version was is satisfactory (Cronbach’s α = 0.78) (31).

#### Insomnia Severity Index (ISI)

The index contains 7 items evaluating the insomnia subtype and daytime dysfunction due to sleep difficulties over the past two weeks. Responses were made on a 5-point Likert scale (0 = not at all, 4 = very severe), ranging from 0 to 28 (32, 33). Chung KF et al. reported the Chinese version of ISI with acceptable internal reliability (Cronbach’s α = 0.83) (34).

#### Pittsburgh Sleep Quality Index (PSQI)

The index is composed of 19 individual items which assesses sleep quality and disturbances over the past 1 month. The 19 items are divided into seven components, including subjective sleep quality, sleep latency, sleep duration, habitual sleep efficiency, sleep disturbances, use of sleeping medication, and daytime dysfunction. Each item is rated by a 0-3 scale, and the seven component scores are summed to a total score, ranging from 0 to 21 (35). The reliability of the Chinese version is satisfactory (Cronbach’s α = 0.81) (36).

#### Epworth Sleepiness Scale (ESS)

The scale is used to assess subjective daytime sleepiness, including 8 items with a 4-point Likert scale (e.g., 0 = no chance of dozing, 3 = high chance of dozing). The total scores range from 0 to 24. The internal consistency of the Chinese version of ESS was accepted (Cronbach’s α = 0.81) (37, 38).

#### Reduced Morningness-Eveningness Questionnaire (rMEQ)

The questionnaire includes five items evaluating morningness-eveningness, rated on a 5-point-Likert scale. Each item was summed up to generate a total score between 4 and 25. The higher score indicated the stronger morning preference. The rMEQ has been validated in ethnic Chinese population, with good psychometric properties (39, 40). The Chinese version of rMEQ has satisfactory psychometric properties (41).

### Sleep Duration

The participants were asked to indicate the bedtimes and rise times on both weekdays and weekends in the past year. The number of minutes was translated into centesimal system, and added to the number of hours to obtain a metric variable.

### Statistical Procedures

Descriptive characteristics at baseline between the responders and non-responders were investigated (**Table 1**). Categorical variables were reported as frequency (percentages) and continuous variables were reported as means (standard deviations).

**Table 1:** Descriptive characteristics at baseline between responders and non-responders at follow-up.

	Total sample(n = 940)	Responders(N = 902)	Non-responders(N = 38)	p value
	Mean (SD) or N (%)	Mean (SD) or N (%)	Mean (SD) or N (%)	
**Socio-demographics**				
Age, years^a^	19.1 (0.9)	19.1 (0.9)	19.0 (0.9)	0.61
Gender, female^b^	594 (63.2)	573 (63.5)	21 (55.3)	0.30
Single child^c^	112 (11.9)	102 (11.3)	10 (26.3)	0.01
Parental education level (Bachelor’s degree or above)^c^	89 (9.5)	85 (9.4)	4 (10.5)	0.78
Maternal education level (Bachelor’s degree or above)^c^	54 (5.7)	50 (5.5)	4 (10.5)	0.35
Paternal migrant workers^b^	469 (49.9)	452 (50.1)	17 (44.7)	0.52
Maternal migrant workers^b^	362 (38.5)	346 (38.4)	16 (42.1)	0.64
Family income (< 5000yuan/month)^b^	371 (39.5)	358 (39.7)	13 (34.2)	0.50
**Lifestyle practices and health conditions**				
BMI (≥ 25kg/m2)^c^	62 (6.6)	59 (6.5)	3 (7.9)	0.73
Habitual napping^d^	894 (95.1)	858 (95.1)	36 (94.7)	0.71
Habitual snoring^b^	143 (15.2)	137 (15.2)	6 (15.8)	0.92
Boarding in school^c^	928 (98.7)	890 (98.7)	38 (100)	1.00
TV/Internet (≥ 3 h/day)^b^	348 (37.0)	333 (36.0)	15 (39.5)	0.75
Tobacco smoking^d^	16 (1.7)	15 (1.7)	1 (2.6)	0.49
Alcohol drinking^b^	443 (47.1)	423 (46.9)	20 (52.6)	0.49
Chronic medical conditions^d^	22 (2.3)	20 (2.2)	2 (5.3)	0.22
Low study pressure Low perceived study pressure^b^	315 (33.5)	300 (33.3)	15 (39.5)	0.43
High interest in study High perceived interest in study^b^	256 (27.2)	243 (26.9)	13 (34.2)	0.32

Values are percentages for categorical variables, means for continuous variables and p value. BMI, Body-Mass Index; SD, standard deviation.

^a^p-value calculated using the independent-samples t-test; ^b^p-value calculated using the Chi-square test; ^c^p-value calculated using the continuity correction test; ^d^p -value calculated using the fisher’s exact test.

A linear regression analysis was performed to identify the association of MPAB (the total score of MPIQ) with mental distress (the total score of BDI and SAS), sleep disturbances (the total score of ISI, PSQI, and ESS), and sleep patterns (the total score of rMEQ, weekday sleep duration, and weekend sleep duration) at baseline. Separate eight models were built respectively for the above variables. Independent variables were the total score of MPIQ. Age and gender were controlled by enter method (**Table 2**).

**Table 2:** Cross-sectional associations of MPAB with mental distress, sleep disturbances, and sleep patterns at baseline.

		MPAB at baseline	
		Crude β ± SE	Adjusted^a^ β ± SE
Mental distress	BDI score	0.31 ± 0.03 ***	0.31 ± 0.03 ***
	SAS score	0.26 ± 0.02 ***	0.25 ± 0.02 ***
Sleep disturbances	PSQI score	0.04 ± 0.01 ***	0.04 ± 0.01 ***
	ISI score	0.11 ± 0.01 ***	0.11 ± 0.01 ***
	ESS score	0.09 ± 0.01 ***	0.08 ± 0.01 ***
Sleep patterns	rMEQ score	-0.01 ± 0.01 ** -0.014 ± 0.005 **	-0.02 ± 0.01 ** -0.015 ± 0.006 **
	weekday sleep duration	-0.002 ± 0.003	-0.001 ± 0.031
	weekend sleep duration	0.005 ± 0.004	0.004 ± 0.043

Values are linear regression coefficients.

^a^Analyses were adjusted for age and gender at baseline (enter method) at baseline. **P < 0.01; ***P < 0.001.N = 940.

MPAB, Mobile Phone Addictive Behaviors; BDI, Beck Depression Inventory; SAS, Zung Self-Rating Anxiety Scale; PSQI, Pittsburgh Sleep Quality Index; ISI, Insomnia Severity Index; ESS, Epworth Sleepiness Scale; rMEQ, reduced Morningness-Eveningness Questionnaire; SE, standard error.

The analysis of Pearson rank correlation matrix was conducted to show the correlation between MPAB at baseline and other main variables studied at baseline and follow-up (**Tables S1** and **S2**). All statistical analyses were conducted with the SPSS statistical software (IBM SPSS Statistics., Chicago, IL).

In addition, structural equation modeling was performed to determine the longitudinal associations (the lagged effects) of MPAB with mental distress, sleep disturbances and sleep patterns. Wald tests were performed to identify the significance of statistical differences between the lagged coefficients for each model. Goodness-of-fit tests were assessed by the following fit indices in a single linear regression: Chi-square, χ^2^/df, Goodness-of-fit index (GFI), adjusted goodness-of-fit index (AGFI), comparative fit index (CFI), and the root mean square error of approximation (RMSEA). The maximum likelihood method was used as model estimator. Models were adjusted for age and sex. The cross-lagged models were conducted by AMOS 22.0 (IBM SPSS Statistics., Chicago, IL). Statistical significance was set at p < 0.05 (2-tailed).

## Results

Characteristics between responders and non-responders of the study sample are presented in **Table 1**. There were 902 (95.9%) participants responding at baseline. Those responders had a lower rate of singleton than the non-responders at baseline. No significant difference was found in other socio-demographics, lifestyle practices and health conditions between responders and non-responders.

**Table 2** presents the cross-sectional associations of MPAB with mental distress, sleep disturbances and sleep patterns at baseline. MPAB at baseline was significantly positively associated with the outcomes of BDI, SAS, PSQI, ISI and ESS (range, β 0.04–0.31), whereas the total score of rMEQ was significantly negatively associated with MPAB at baseline (adjusted β = -0.01). However, there is no significant relationship between MPAB and sleep duration. After adjustment, these associations at baseline remained statistically significant (range, adjusted β 0.04–0.31), including for the outcome of rMEQ (adjusted β = -0.02).

**Figure 1:**
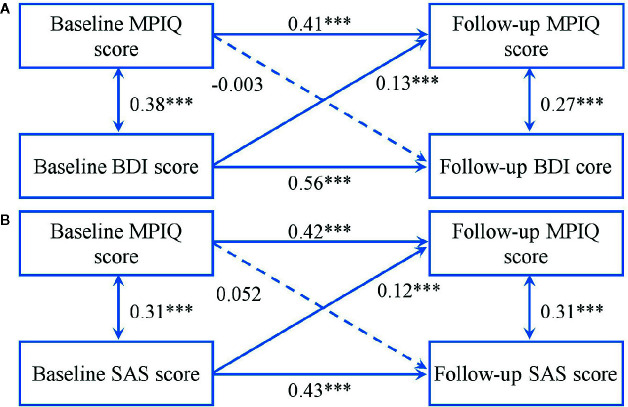
Cross-lagged models of MPA with BDI score **(A)** and SAS score **(B)**. Models were adjusted for age and sex. Standardized β coefficients are stated in longitudinal associations at baseline and follow-up. ***P < 0.001. Solid lines present statistically significant associations and dashed lines present nonsignificant associations. MPAB, Mobile Phone Addictive Behaviors; BDI, Beck Depression Inventory; SAS, Zung Self-Rating Anxiety Scale.

**Figure 1** shows the results of cross-lagged models between MPAB and mental distress. The two models showed an acceptable fit (BDI: χ^2^ = 29.23, χ^2^/df = 5.85, P < 0.001; GFI = 0.99, AGFI = 0.95, CFI = 0.97, RMSEA = 0.07; SAS: χ^2^ = 30.29, χ^2^/df = 6.06, P < 0.001; GFI = 0.99, AGFI = 0.95, CFI = 0.96, RMSEA = 0.07). Results indicated that, a higher total score of BDI and SAS predicted a greater likelihood of subsequent MPAB, but not vice versa.

**Figure 2:**
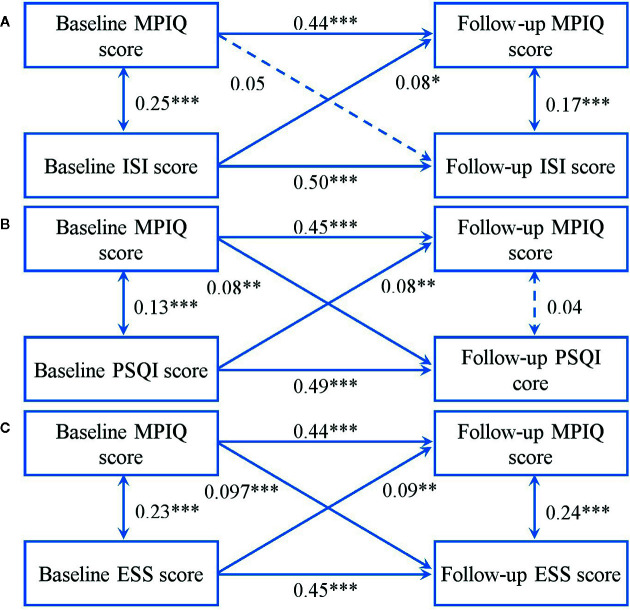
Cross-lagged models of MPA with ISI score **(A)**, PSQI score **(B)**, and ESS score **(C)**. Models were adjusted for age and sex. Standardized β coefficients are stated in longitudinal associations at baseline and follow up. *P < 0.05; **P < 0.01; ***P < 0.001. Solid lines present statistically significant associations and dashed lines present nonsignificant associations. MPAB, Mobile Phone Addictive Behaviors; PSQI, Pittsburgh Sleep Quality Index; ISI, Insomnia Severity Index; ESS, Epworth Sleepiness Scale.

**Figure 2** indicates the results of cross-lagged models between MPAB and sleep disturbances. Fit indexes for each model were acceptable (ISI: χ^2^ = 29.50, χ^2^/df = 5.90, P < 0.001; GFI = 0.99, AGFI = 0.95, CFI = 0.96, RMSEA = 0.07; PSQI: χ^2^ = 28.97, χ^2^/df = 5.71, P < 0.001; GFI = 0.99, AGFI = 0.95, CFI = 0.96, RMSEA = 0.07; ESS: χ^2^ = 27.09, χ^2^/df = 5.42, P < 0.001; GFI = 0.99, AGFI = 0.96, CFI = 0.97, RMSEA = 0.07). We found that a higher total score of ISI at baseline predicted higher total score of MPIQ at follow-up, but no associations in the opposite direction. It also delineated the bidirectional longitudinal relationships between MPAB and the total score of PSQI and ESS.

**Figure 3:**
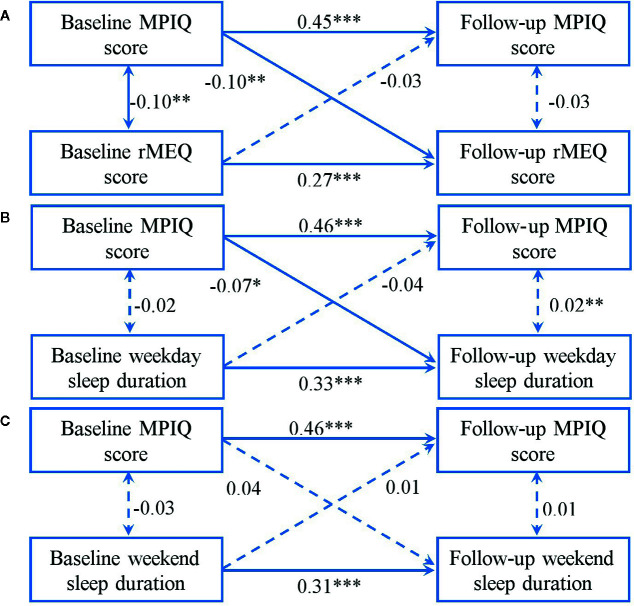
Cross-lagged models of MPAB with rMEQ score **(A)**, weekday sleep duration **(B)**, and weekend sleep duration **(C)**. Models were adjusted for age and sex. Standardized β coefficients are stated in longitudinal associations at baseline and follow-up. *P < 0.05; **P < 0.01; ***P < 0.001. Solid lines present statistically significant associations and dashed lines present nonsignificant associations. MPAB, Mobile Phone Addictive Behaviors; rMEQ, reduced Morningness-Eveningness Questionnaire.

**Figure 3** presents cross-lagged models between MPAB and sleep patterns. The results showed an acceptable fit (rMEQ: χ^2^ = 32.36, χ^2^/df = 6.47, P < 0.001; GFI = 0.99, AGFI = 0.95, CFI = 0.92, RMSEA = 0.08; weekday sleep duration: χ^2^ = 26.70, χ^2^/df = 5.34, P < 0.001; GFI = 0.99, AGFI = 0.96, CFI = 0.94, RMSEA = 0.07; weekend sleep duration: χ2 = 28.68, χ2/df = 5.74, GFI = 0.99, AGFI = 0.96, CFI = 0.93, RMSEA = 0.07). It was shown that a higher score of MPIQ at baseline predicted a subsequent lower total score of rMEQ and shorter weekday sleep duration. Nonetheless, no relationship between MPAB and weekend sleep duration across time was significant in either direction.

**Tables S1** and **S2** delineate the cross-sectional and long-term Spearman correlation coefficients of MPAB at baseline with mental distress, sleep disturbances and sleep patterns. At the both stages, significant and positive correlations were found between MPAB and all the outcomes of mental distress and sleep disturbances (range, r 0.13–0.37), while the severity of MPAB showed a negative correlation with the total score of rMEQ (baseline: r = -0.09; follow-up: r = -0.12). In addition, there is a significant correlation between MPAB and weekday sleep duration at follow-up (r = -0.11).

## Discussion

The main findings of this study are as follows: (1) the severity of depression and anxiety at baseline were predictive factors of MPAB at one-year follow-up, but not in the opposite direction. (2) a severe level of insomnia may work as an important reminder of MPAB, while there were bidirectional relationships of MPAB with poorer sleep quality and stronger daytime sleepiness. (3) later chronotype preference and shorter weekday sleep duration were negative consequences of MPAB, but not causes of it.

This study showed people with depression and/or anxiety were more likely to get involved in MPAB, comparing to those without mental problems, which were consistent with previous finding (24, 42, 43). Depressive individuals were found to achieve decreased pleasure from social interactions and increased sensitivity to social rejection, but a tendency of more frequent usage of social media (44). Frequent mobile devices use as a kind of avoidance-coping strategy seems to bring forward a feasible substitution of discomforting face-to-face contact with the social situations for them (4, 45). In fact, on account of the social enhancement model that the rich get richer, the poor get poorer, those with psychological distress are not only unable to enjoy the advantage of technology, but also more likely to be trapped into problematic media use (43, 46). Such avoidance-coping tended to result in subsequent troubles of life events and further fueled the dependency on online activities (45, 47). Instead of the preceding escapism motivation, rely on mobile phones could also be another form of interpersonal dependency, which was closely related to anxiety symptoms (48). For example, a group called “Fear-Of-Missing-Out (FOMO)” can be understood to be so anxious around missing out on rewarding experiences that stay in ever close contact. A high level of perception of social stress was proved to be positively correlated with MPA (3). Moreover, neuroticism, which contributed to increasing emotional reaction, tends to make more efforts to maintain the social relationship by check text-message repeatedly on mobile phones due to bad performances in face-to-face communication (49).

However, MPAB at baseline cannot predict the depression and/or anxiety symptoms at follow-up in this study. It is inconsistent with the result of the previous studies, which suggested the reciprocal relationship between MPA and mental problems (15, 16). In fact, it has been reported that rely on mobile phones could alleviate negative emotions when facing depression or loneliness (43). We speculated online supports obtained from MPAB may provide temporary but not necessarily short-time relief to people suffering from the disturbing events, despite its obvious drawbacks. Finally, social isolation and subjective perception of social stress due to MPAB may further aggravate depression and anxiety symptoms in a further way (4, 43). Another possible explanation of the controversy results is that the severity of depression and/or anxiety resulting from MPAB could not necessarily evolve over time. In addition, different measurements of the severity of MPAB may result in the inconsistent use of the term MPAB, and have produced variant results regarding the link between mental stress and MPAB.

Furthermore, we found that insomnia symptoms, poorer sleep quality and stronger daytime sleepiness promoted the development of MPAB. The former factor worked as a predictor, while the latter two exacerbate the severity of MPAB in the opposite direction. The literature in the field between behavior addiction and sleep problems was still limited to clarify exactly this result. However, we found a common trajectory between sleep disturbance and alcohol and other drug that of childhood sleep difficulties and insufficient sleep, earlier initiation into substance use and misconduct related to substance use, such as drunk driving and substance overuse (50). Thus, we conjectured that incipient sleep problems promote the habit of repetitive mobile phone use at night and result in erratic sleep/wake behavior. Further, more variable bedtime and insomnia symptoms were found to contribute to poor self-regulation and impulsivity trait, which increased the risk of addiction behavior (51). On the other hand, using mobile devices after light-out also contributes to negative outcomes of sleep quality or quantity. There are three possible mechanisms that were widely accepted: (1) media use displaces sleep or other activities that promote good sleep; (2) media use causes increases in physiological arousal; and (3) bright light exposure at evening delays the circadian rhythm by a reduction in melatonin secretion (52).

Another finding of the study is that the high level of MPA may bring about a later chronotype and shorter weekday sleep duration. Indeed, it could be difficult for individuals with MPAB to put down mobile phones at night. Firstly, addicted people show an explicit preference for immediate rewards but not delayed and better ones by the reason of the “delayed discount effect” (53). In other words, these with MPAB are more likely to fail to resist the urge on the pleasure brought from the mobile phone resulting in postponing bedtime even if they have an important schedule tomorrow morning. Next, the underestimation of time led by the increase in DAergic activity plays an important role in erratic and later sleep times (54). The other directly-related reason for the shift to a later chronotype could probably be that light-emitted screens suppress melatonin secretion at bedtimes and delayed the circadian rhythm (52, 55). Besides, shorter weekday sleep duration, which was consistent with the previous studies, could be explained that mobile phone overuse displaces sleep duration (7). However, there is no significant relationship between MPAB and weekend sleep duration, which may be due to common habits of weekend sleep compensation among young people.

There are several limitations in the current study. First, we only recruited medical undergraduates from the same college. Assuming that young individuals from more schools and majors can be conducted as a larger sample, the findings could be better generalized for the general population. Secondly, only two time points were assessed in this study, which may influence a comprehensive understanding of the associations between mobile phone addiction behaviors and various outcomes, especially mental distress. Additionally, some notable confounding factors (e.g., some important life events, the studying pattern of medical students in China) were not considered in the study. For example, Chinese medical students are under great pressure on study, which may lead to shorter sleep duration. However, another study of our team researched sleep patterns of technical college students and found average sleep duration of 7.60 h on weekdays and 9.25 h on weekends, which are similar to our outcomes (22). The mean sleep duration in our study was 7.67 h (SD, 0.96) on weekdays and 8.92 h (SD, 1.25) among the medical students. Next, mobile application preference and other related questions did not be investigated so that we failed to provide clear confirmation of the usage mode for the addictive people to support the discussion surrounding the topic. Fourthly, the study has limitations in the selection of questionnaires. MPIQ was developed in the “mobile phone era”, while most people use smartphones at present. It may result in bias. Nevertheless, as described by the designer of MPIQ, mobile phones provide some traditional functions, such as calling, alarm clocks, cameras, diaries, and phone books, which are also essential and commonly used functions of smartphones (26). Further, we found a study, published in 2020, reported the Chinese version of MPIQ with acceptable internal reliability and validity among college students with smartphones (56). Besides, we used the first version of SAS instead of the latest version. Finally, we assessed all the variables by self-reporting. Self-reported data may lead to recall bias and reduce reliability and validity. More semi-structured interviews or objective measurement tools could be used for further exploration in future researches.

## Conclusion

In summary, anxiety, depression and insomnia symptoms play a significant role in the promotion of MPAB among young adults. The negative effect of MPAB was shown by the preference for later chronotype and longer weekday sleep duration in the study. We also found the bidirectional relationship between MPAB and several sleep disturbances, including sleep quality and excessive daytime sleepiness. Therefore, we should strengthen the early intervening measure for mental health problems to prevent the addiction behaviors related to mobile phones among young people. Besides, the adverse impact of excessive using mobile phones should be focused around the campus.

## Data Availability Statement

The datasets generated for this study will not be made publicly available because other authors are still using the datasets to write articles. Requests to access the datasets should be directed to the corresponding author.

## Ethics Statement

The studies involving human participants were reviewed and approved by Medical ethics committee, Nanfang Hospital, Southern Medical University. The participants provided written informed consent to participate in this study.

## Author Contributions

YK: Formal analysis, Writing—original draft, Investigation. SL: Conceptualization, Data curation, Formal analysis, Writing—review and editing, Supervision. BX: Recruitment, Methodology, Data curation, Methodology. LY, LL, LX, WZ: Recruitment, Data curation, Writing—review and editing. JZ: Conceptualization, Methodology, Writing—review and editing, Supervision. BZ: Funding acquisition, Conceptualization, Methodology, Writing—review and editing, Supervision.

## Funding

This study is funded by the President Foundation of Nanfang Hospital, Southern Medical University (2019Z014), Project of Guangzhou Philosophy and Society Development (2018GZGJ58), and National Natural Science Foundation of China (81901348).

## Conflict of Interest

The authors declare that the research was conducted in the absence of any commercial or financial relationships that could be construed as a potential conflict of interest.

## Abbreviations

MPAB, Mobile phone addiction behaviors; BMI, body-mass index; MPIQ, Mobile Phone Involvement Questionnaire; BDI, Beck Depression Inventory; SAS, Zung Self-Rating Anxiety Scale; ISI, Insomnia Severity Index; PSQI, Pittsburgh Sleep Quality Index; ESS, Epworth Sleepiness Scale; rMEQ, reduced Morningness-Eveningness Questionnaire; SD, standard deviation; SE, standard error.
