# Supplementary material for: Testing the Bidirectional Associations of Mobile Phone Addiction Behaviors With Mental Distress, Sleep Disturbances, and Sleep Patterns: A One-Year Prospective Study Among Chinese College Students
Source: Front Psychiatry. 2020 Jul 17;11:634. doi: 10.3389/fpsyt.2020.00634 (PMC7379372; doi:10.3389/fpsyt.2020.00634)
Supplement: Supplementary file 1 [file Table_1.docx]

Table S1 Correlation matrix of MPAB at baseline with mental distress, sleep disturbances and sleep patterns at baseline

|  | 1 | 2 | 3 | 4 | 5 | 6 | 7 | 8 | 9 |
| --- | --- | --- | --- | --- | --- | --- | --- | --- | --- |
| 1. MPIQ score | - |  |  |  |  |  |  |  |  |
| 2. BDI score | 0.38  *** | - |  |  |  |  |  |  |  |
| 3. SAS score | 0.34  *** | 0.58  *** | - |  |  |  |  |  |  |
| 4. PSQI score | 0.14  *** | 0.40  *** | 0.23  *** | - |  |  |  |  |  |
| 5. ISI score | 0.25  *** | 0.50  *** | 0.37  *** | 0.64  *** | - |  |  |  |  |
| 6. ESS score | 0.25  *** | 0.34  *** | 0.32  *** | 0.18  *** | 0.32  *** | - |  |  |  |
| 7. rMEQ score | -0.09  ** | -0.13  *** | -0.06 | -0.09  ** | -0.09  ** | -0.06  * | - |  |  |
| 8. weekday sleep duration | -0.02 | -0.09  ** | -0.02 | -0.02 | -0.03 | -0.01 | 0.06 | - |  |
| 9. weekend sleep duration | 0.04 | 0.03 | 0.03 | 0.05 | 0.01 | 0.06  * | -0.17  *** | 0.39  *** | - |

Values are Pearson correlation coefficients. *P < 0.05; **P < 0.01; ***P < 0.001.N = 940.

MPAB, Mobile Phone Addictive Behaviors; MPIQ, Mobile Phone Involvement Questionnaire; BDI, Beck Depression Inventory; SAS, Zung Self-Rating Anxiety Scale; PSQI, Pittsburgh Sleep Quality Index; ISI, Insomnia Severity Index; ESS, Epworth Sleepiness Scale; rMEQ, reduced Morningness-Eveningness Questionnaire.

Table S2 Correlation matrix of MPAB at baseline with mental distress, sleep disturbances and sleep patterns at follow-up

|  | 1 | 2 | 3 | 4 | 5 | 6 | 7 | 8 | 9 |
| --- | --- | --- | --- | --- | --- | --- | --- | --- | --- |
| 1. MPIQ score | - |  |  |  |  |  |  |  |  |
| 2. BDI score | 0.21  *** | - |  |  |  |  |  |  |  |
| 3. SAS score | 0.20  *** | 0.62  *** | - |  |  |  |  |  |  |
| 4. PSQI score | 0.14  *** | 0.41  *** | 0.27  *** | - |  |  |  |  |  |
| 5. ISI score | 0.17  *** | 0.56  *** | 0.42  *** | 0.60  *** | - |  |  |  |  |
| 6. ESS score | 0.21  *** | 0.45  *** | 0.38  *** | 0.27  *** | 0.37  *** | - |  |  |  |
| 7. rMEQ score | -0.13  *** | -0.12  *** | -0.10  ** | -0.14  *** | -0.15  *** | -0.09  * | - |  |  |
| 8. weekday sleep duration | -0.10  ** | 0.04 | 0.11  ** | 0.11  ** | 0.04 | -0.04 | 0.04 | - |  |
| 9. weekend sleep duration | 0.05 | 0.07  * | 0.07  * | 0.12  *** | 0.08  * | 0.03 | -0.14  *** | 0.28  *** | - |

Values are Pearson correlation coefficients. *P < 0.05; **P < 0.01; ***P < 0.001.N = 902.

MPAB, Mobile Phone Addictive Behaviors; MPIQ, Mobile Phone Involvement Questionnaire; BDI, Beck Depression Inventory; SAS, Zung Self-Rating Anxiety Scale; PSQI, Pittsburgh Sleep Quality Index; ISI, Insomnia Severity Index; ESS, Epworth Sleepiness Scale; rMEQ, reduced Morningness-Eveningness Questionnaire.
